# Imaging Characteristics and Endovascular Treatment of Brain Arteriovenous Malformations Mainly Fed by the Posterior Cerebral Artery

**DOI:** 10.3389/fneur.2020.609461

**Published:** 2021-01-27

**Authors:** Kun Hou, Chao Li, Han Su, Jinlu Yu

**Affiliations:** ^1^Department of Neurosurgery, The First Hospital of Jilin University, Changchun, China; ^2^Department of Neurology, The First Hospital of Jilin University, Changchun, China

**Keywords:** posterior cerebral artery, brain arteriovenous malformation, endovascular treatment, imaging characteristics, posterior choroidal artery

## Abstract

**Background:** A BAVM that is mainly supplied by the posterior cerebral artery (PCA) lies deeply in the middle of the bilateral posterior hemispheres. Few studies have investigated the imaging characteristics and endovascular treatment (EVT) of brain arteriovenous malformations (BAVMs) in this area.

**Methods:** A retrospective study was performed for patients who were diagnosed with PCA-BAVMs from January 2015 to December 2019. The PCA-BAVMs were divided into type I and type II according to their feeding arteries. Type I PCA-BAVMs were supplied by the posterior choroidal artery (PchA) from the PCA. They could be further subdivided into type Ia and type Ib. Type II PCA-BAVMs were supplied by the temporal or occipital branch from the PCA. They could also be further subdivided into type IIa and IIb. Targeted embolization of the risk factors was the main aim of EVT.

**Results:** Forty-two patients were identified, with age ranging from 6 to 63 years. Twenty-four cases belonged to type I (57.1%, 24/42), including 6 Ia cases and 18 Ib cases. Eighteen cases belonged to type II (42.9%, 18/42), including 7 IIa cases and 11 IIb cases. Immediate complete or nearly complete embolization was achieved in 17 (40.5%, 17/42) cases. Partial embolization was achieved in 25 (59.5%, 25/42) cases. Two (4.8%, 2/42) patients experienced intraoperative or postoperative bleeding. The GOS scores at discharge were 3, 4, and 5 in 2 (4.8%, 2/42), 2 (4.8%, 2/42), and 38 (90.4%, 38/42) cases, respectively. There was no statistical difference between patients in type I and type II groups regarding age, BAVM rupture, SM grade, immediate extent of obliteration, and prognosis. Deep venous drainage was more common in patients of the type I group (*P* < 0.001).

**Conclusions:** Our classification of the PCA-BAVMs was based on the segmentation of the PCA, which is a reasonable approach and could guide the strategy of EVT. EVT is a reasonable option for the PCA-BAVMs. The main aim of EVT is to secure the weak structures. A targeted EVT aimed at the ruptured part of the BAVM can reduce the risk of early rebleeding.

## Introduction

A brain arteriovenous malformation (BAVM) is a common congenital vascular disease that belongs to the abnormal nidus between arteries and veins, which lacks an intervening capillary network and is characterized by a complex, tangled web of abnormal vessels (1, 2). Moreover, BAVMs recruit blood supply from their neighboring arteries and drain to the adjacent veins (3). BAVMs in different positions may have different characteristics (4).

A BAVM that is mainly supplied by the posterior cerebral artery (PCA) lies deeply in the middle of the bilateral posterior hemispheres and near the posterior part of the corpus callosum and the deep cerebral venous system (5). The feeding arteries of the PCA-supplied BAVMs (PCA-BAVMs) are complex, and they often drain to the deep venous system. However, few studies have investigated the angiographic characteristics and endovascular treatment (EVT) for BAVMs in this area. Therefore, in this study, we conducted a retrospective single-center investigation of the patients who were diagnosed with PCA-BAVMs.

## Materials and Methods

A retrospective study was performed for patients who were admitted to The First Hospital of Jilin University diagnosed with BAVMs mainly supplied by the PCA system from January 2015 to December 2019. This study was approved by the institutional ethics committee.

### Inclusion Criteria

(1) The BAVM was located at the supplied area of the PCA, which included posterior part of the callosum, pineal region, medial posterior part of the temporal lobe, and medial part of the occipital lobe. (2) PCA was the main (if not the only) source of blood supply. (3) No previous EVT, open surgery, or radiosurgery was performed before admitting to our institution.

### Classification of the PCA-BAVM

The PCA-BAVMs were divided into the following types according to their feeding arteries.

Type I: supplied by the posterior choroidal artery (PchA) from the PCA. It could be further subdivided into type Ia and type Ib. For type Ia, the PchA was the only source of arterial supply, while type Ib received additional blood supply from other sources, which included the perforating artery from the P1 segment of the PCA, the anterior choroidal artery (AchA), the anterior cerebral artery (ACA), and so on.

**Figure 1:**
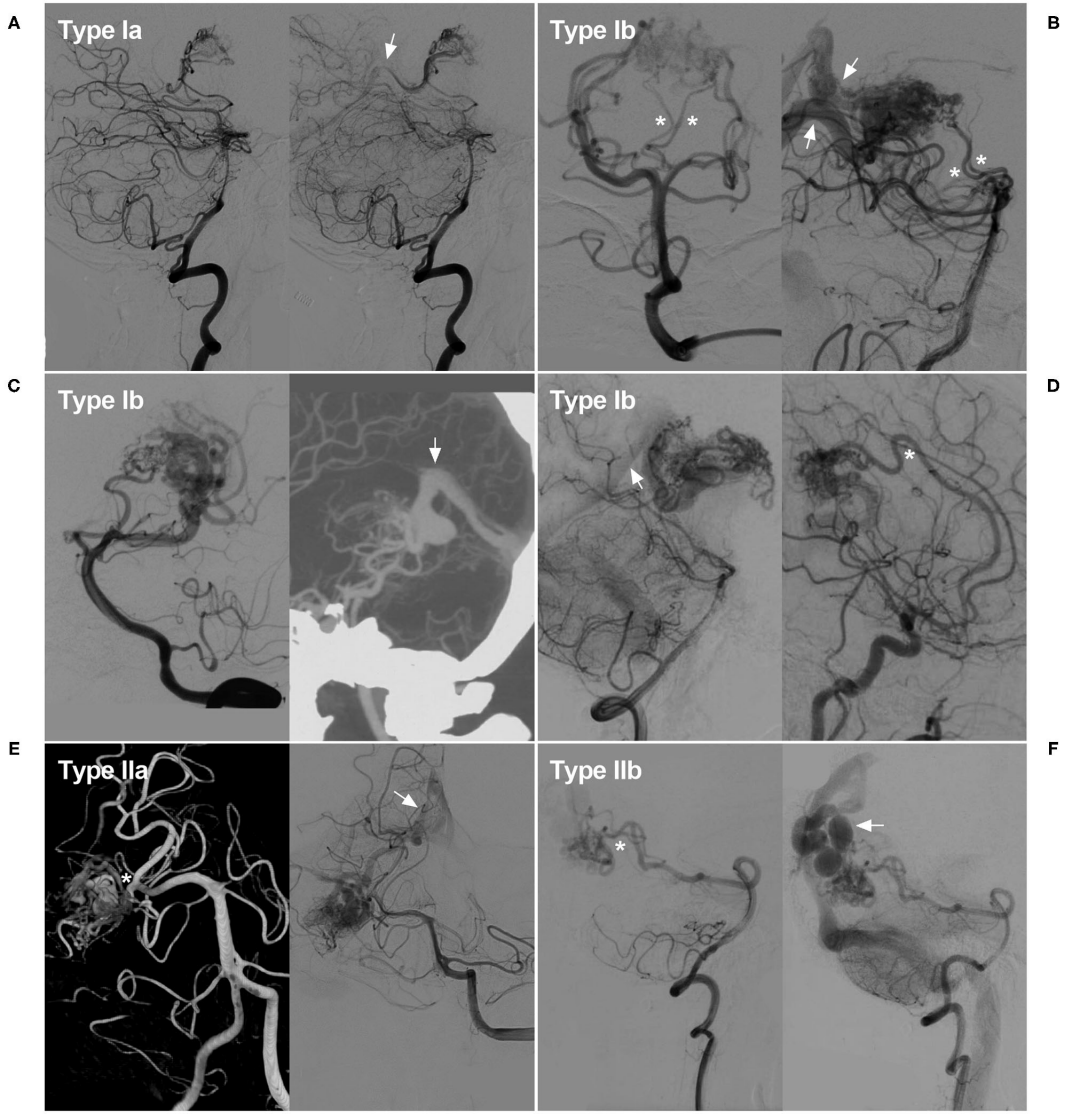
Classification of the PCA-BAVMs. **(A)** Angiogram of the VA in lateral view shows a Type Ia BAVM supplied by the PchA from the PCA only and drained to the deep venous system (arrow). **(B)** Angiogram of the VA in anteroposterior (left) and lateral (right) views shows a type Ib BAVM supplied by the PchA (asterisk) and the proximal perforating branches of the PCA (asterisks). Multiple deep venous drainage (arrows) can be observed. **(C)** Angiogram of the VA in lateral (left) view shows a type Ib BAVM supplied by the PchA and the lateral and medial occipital branches of the PCA. Computed tomography angiography (right) in maximum intensity projection shows the BAVM drains through the deep venous system (arrow). **(D)** Angiogram of the VA (left) and internal carotid artery (right) in lateral view shows a type Ib BAVM supplied by the PchA and ACA (asterisk). Deep venous drainage (arrow) can be observed. **(E)** Angiogram of the VA shows a Type IIa BAVM supplied by the temporal branch (asterisk) of the PCA and drained to the deep venous system (arrow). **(F)** Angiogram of the VA shows a Type IIb BAVM supplied by the medial occipital branch (asterisk) and drained through the cortical veins (arrow). ACA, anterior cerebral artery; BAVM, brain arteriovenous malformation; PCA, posterior cerebral artery; PchA, posterior choroidal artery; VA, vertebral artery.

Type II: supplied by the temporal or occipital branch from the PCA. It could also be further subdivided into type IIa and IIb. Type IIa denoted those located at the proximal segment of the PCA and supplied by the temporal branch (including the anterior and posterior temporal arteries). While type IIb denoted those located at the distal segment of the PCA and supplied by the occipital branch of the PCA (including the lateral and medial occipital branches). Some illustrative cases are presented in Figure 1 to expound the classification system.

### Scheme and Strategy of EVT

For the EVT of BAVMs, we preferred the transarterial approach. Transvenous approach was the last resort. For aneurysms on the feeding arteries, we used the Echelon (Medtronic, Irvine, California, USA) microcatheter for coiling. To obliterate the BAVM or aneurysm near the BAVM, we used Marathon (Medtronic, Irvine, California, USA) microcatheter to cast Onyx. To avoid Onyx reflux, the proximal feeding artery could be partially coiled, creating the so-called pressure cooker effect.

The specific strategy adopted for a ruptured BAVM was determined by the ruptured or bleeding structure. For a ruptured aneurysm on the feeding artery, simple coiling or stent-assisted coiling was preferred. When the aneurysm was near the BAVM, parent artery occlusion (PAO) was preferred. For a ruptured intranidal aneurysm, the EVT was targeting the BAVM compartment harboring the ruptured intranidal aneurysm. If there were no clear risk factors, the aim of EVT was reducing the blood flow of the ruptured BAVM (6). For an unruptured BAVM, identification and management of the weak structures, such as aneurysm on the feeding artery or dilated structure in the BAVM, were of utmost importance. If no weak structures were identified or the venous drainage was patent, a wait-and-see regimen would be adopted. For the patients with obstructed venous drainage, we would like to embolize the BAVM nidus to reduce venous volume load.

After EVT of the risk factors, if the venous drainage was patent, a wait-and-see regimen could be adopted. Otherwise, another session of EVT, open surgery, or radiosurgery would be recommended to the patient.

### Follow-Up and Evaluation of Outcome

The extent of obliteration was classified into complete/nearly complete obliteration and partial obliteration. Complete or nearly complete obliteration was defined as invisible or near-invisible nidus and venous drainage. Partial obliteration was defined as decrease in size but still visible. Complications, further treatment, and Glasgow Outcome Scale (GOS) score at discharge and follow-up were also recorded.

### Statistical Analysis

GraphPad Software (LLC, San Diego, USA) was used to perform statistical analysis. Continuous variables were expressed as the mean ± standard deviation. The Chi-square or Fisher's exact test was used to analyze count or categorical data. *P* < 0.05 was considered with statistical significance.

## Results

### General Information

Forty-two patients were identified, with age ranging from 6 to 63 years (mean 31.5 ± 14.7 years). Fifteen patients were males (35.7%, 15/42). The BAVMs were unruptured in 8 (19.0%, 8/42) patients, of whom five were admitted for headache and three were admitted for epilepsy. The remaining 34 (81.0%, 34/42) patients presented with intracranial bleeding, including two patients with subarachnoid hemorrhage (SAH), two patients with SAH and intracerebral hematoma (IH), nine patients with IH, 16 patients with intraventricular hemorrhage (IVH), and five patients with IH + IVH. For the ruptured cases, the Hunt-Hess grades were I, II, and III in 26, 7, and 1 patient, respectively.

### Imaging Characteristics

#### Classification of PCA-BAVMs

Twenty-four cases belonged to type I (57.1%, 24/42), including 6 Ia cases and 18 Ib cases. Eighteen cases belonged to type II (42.9%, 18/42), including 7 IIa cases and 11 IIb cases.

#### Sizes and Spetzler-Martin (SM) Grades of the BAVMs

The maximum size of BAVMs ranged from 0.8 to 9 cm (4.0 ± 1.8 cm). Five cases belonged to SM grade 1 (11.9%, 5/42), 14 cases belonged to grade 2 (33.3%, 14/42), 13 cases belonged to grade 3 (31.0%, 13/42), and 10 cases belonged to grade 4 (23.8%, 10/42). Four (9.5%, 4/42) cases were identified with feeding artery or intranidal aneurysms.

#### Venous Drainage

Thirteen cases had superficial venous drainage (31.0%, 13/42), 26 cases had deep venous drainage (61.9%, 26/42), and 3 cases (7.1%, 3/42) had both superficial and deep venous drainage.

The clinical and imaging data of the patients are summarized in Table 1.

**Table 1:** Clinical data of the patients.

Age (years)	Mean	31.5 ± 14.7
	Range	6–63
Female/Male	15/27	
Presentation	Unruptured	8 (19.0%, 8/42)
	Ruptured	34 (81.0%, 34/42)
Hunt-Hess*	I	26
	II	7
	III	1
Nidus Size (cm)	Mean	4.0 ± 1.8
	Range	0.8–9
Spetzler-Martin grade	1	5 (11.9%, 5/42)
	2	14 (33.3%, 14/42)
	3	13 (31.0%, 13/42)
	4	10 (23.8%, 10/42)
Classification	Type I	24 (57.1%, 24/42)
	Type II	18 (42.9%, 18/42)
Associated aneurysm	4 (9.5%, 4/42)	
Venous drainage	Superficial vein drainage	13 (31.0%, 13/42)
	Deep vein drainage	26 (61.9%, 26/42)
	Both	3 (7.1%, 3/42)

* *Hunt-Hess grade for ruptured cases*.

### EVT

Onyx embolization of the BAVMs through the PCA was performed in 33 (78.6%, 33/42) cases, of which two cases used the pressure cooker technique. Onyx embolization of the BAVMs through the ACA was performed in 7 (16.6%, 7/42) cases, of which one case used the pressure cooker technique. Onyx embolization of the BAVMs through the ACA + PCA was performed in 2 (4.8%, 2/42) cases.

Four cases with feeding artery or intranidal aneurysms underwent Onyx embolization of the aneurysms. Some typical cases of EVT are shown in Figures 2–9.

**Figure 2:**
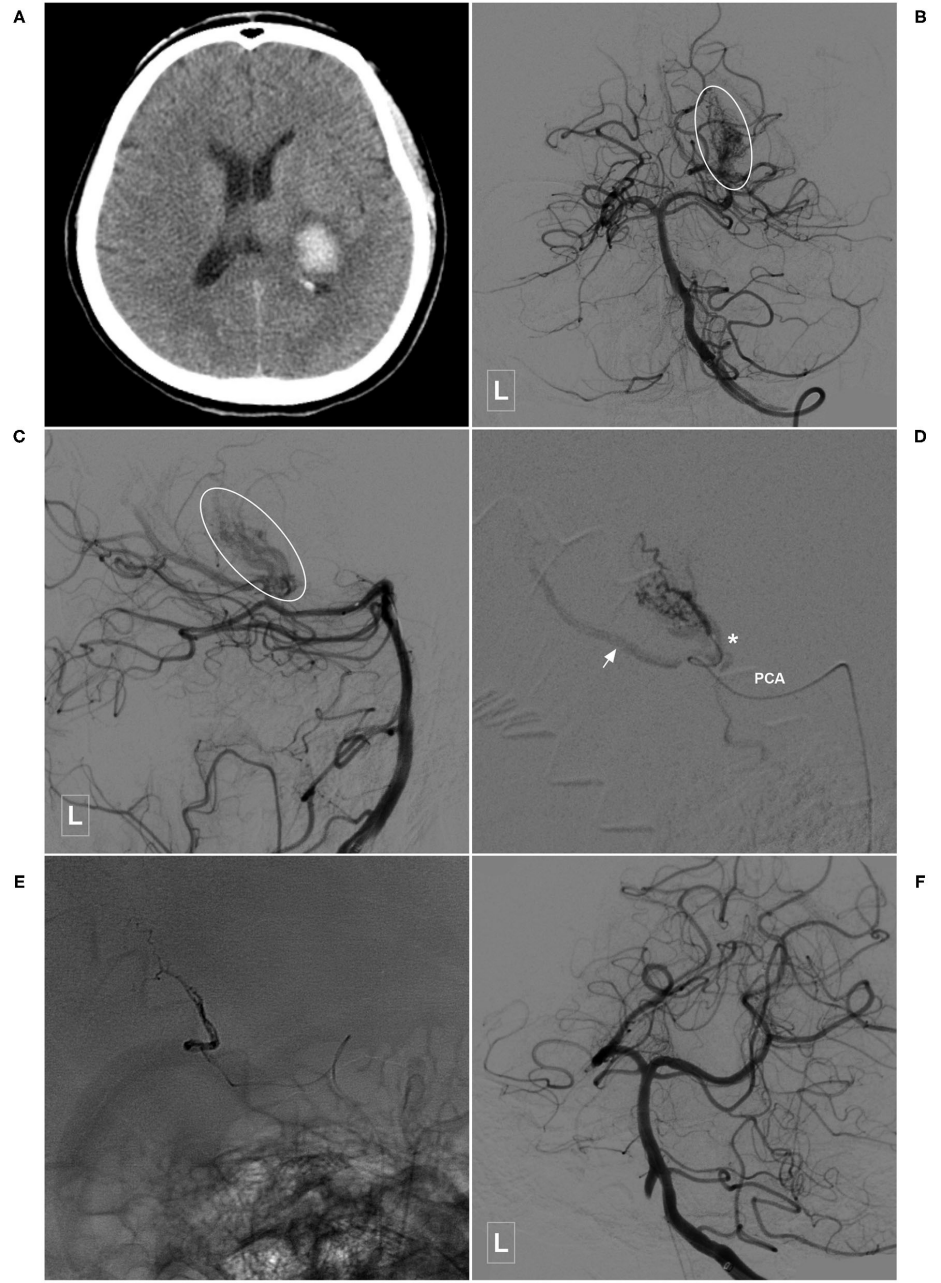
Illustrative case of a Type Ia PCA-BAVM. **(A)** Computed tomography shows hemorrhage at the left thalamus with ventricular extension. **(B,C)** Angiogram of the left VA in the anteroposterior **(B)** and lateral **(C)** views shows a Type Ia PCA-BAVM (encircled area) solely supplied by the PchA. **(D)** Super-selective angiogram of the BAVM. Arrow denotes the draining vein and the asterisk denotes the lateral PchA. **(E)** X-ray of the cranium after embolization shows Onyx casting. **(F)** Angiogram of the left VA shows complete embolization of the BAVM. BAVM, brain arteriovenous malformation; PCA, posterior cerebral artery; PchA, posterior choroidal artery; VA, vertebral artery.

**Figure 3:**
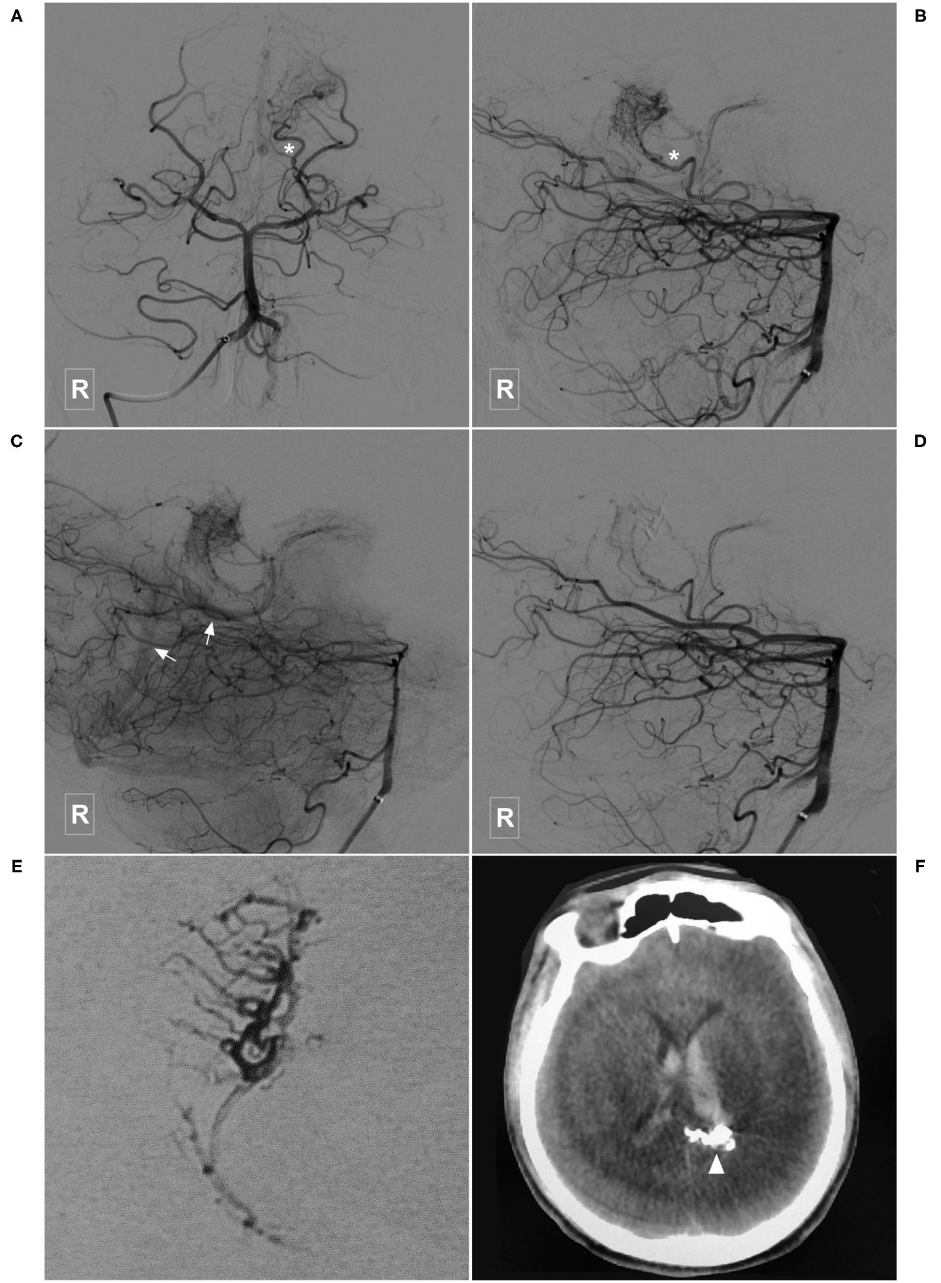
Illustrative case of a Type Ia PCA-BAVM. **(A,B)** Angiogram of the right VA in the anteroposterior **(A)** and lateral **(B)** views shows a BAVM supplied by the lateral PchA (asterisk). **(C)** Angiogram of the left VA in lateral view at venous phase shows that the BAVM drains to the deep venous system (arrow). **(D)** Angiogram of the left VA after embolization shows the complete obliteration of the BAVM. **(E)** X-ray of the cranium shows the Onyx casting in the BAVM. **(F)** Postoperative computed tomography shows Onyx (arrowhead) and intraventricular hemorrhage. BAVM, brain arteriovenous malformation; PchA, posterior choroidal artery; VA, vertebral artery.

**Figure 4:**
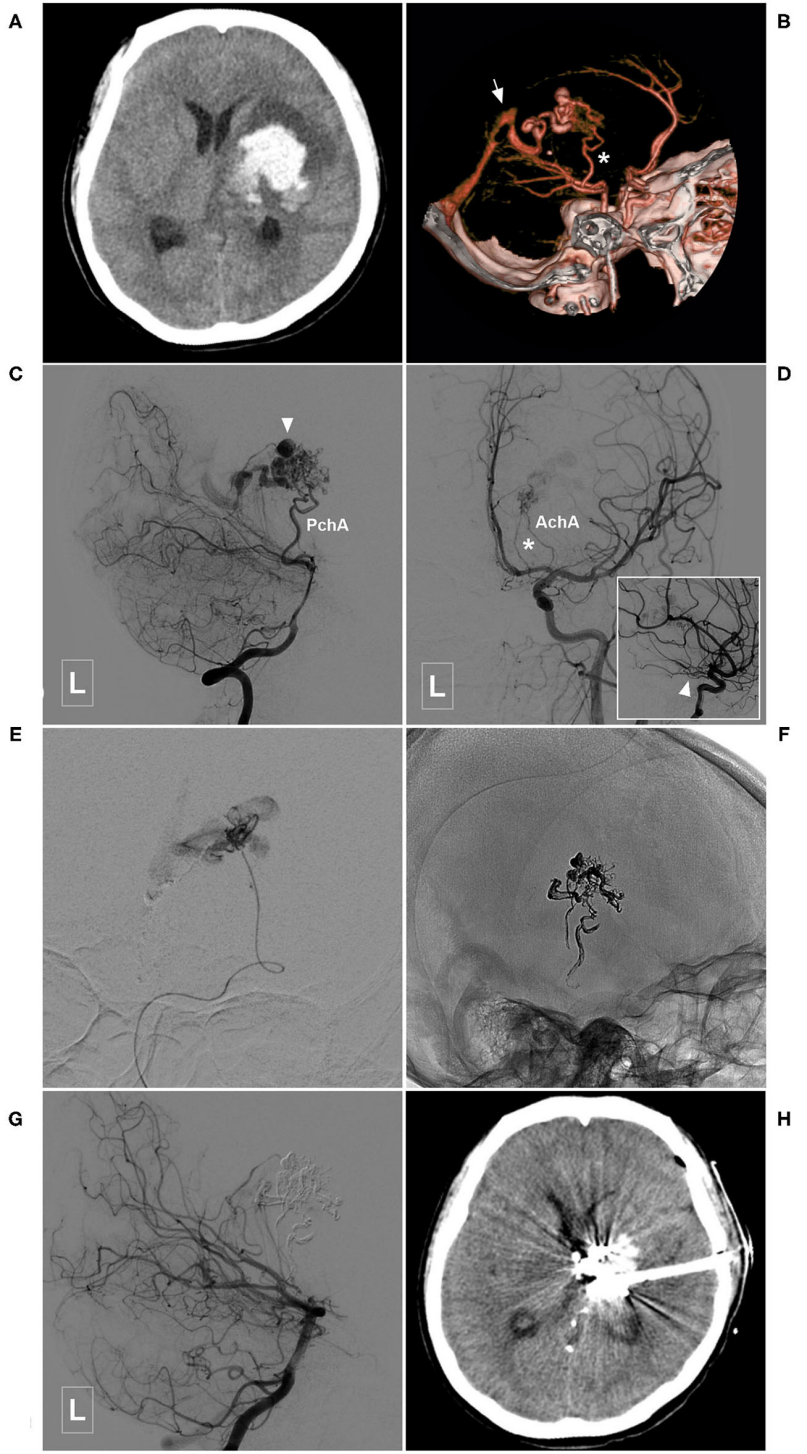
Illustrative case of the Type Ia PCA-BAVM with intranidal aneurysm. **(A)** CT shows hemorrhage at the left thalamus and basal ganglia. **(B)** CT angiography reveals a BAVM fed by the left PchA (asterisk) and drained to the deep venous system (arrow). **(C)** Angiogram of the left VA in lateral view shows that the BAVM is supplied by the PchA and an intranidal aneurysm (arrowhead) is also noted. **(D)** Angiogram of the left internal carotid artery in anteroposterior view shows the left AchA (asterisk) also provides some blood supply to the BAVM. Arrowhead in the square denotes the proximal segment of the AchA. **(E)** Super-selective angiogram of the BAVM. **(F)** X-ray of the cranium after Onyx casting. **(G)** Angiogram of the left VA in lateral view shows complete obliteration of the BAVM. **(H)** CT shows that the hematoma is drained out after embolization of the BAVM. BAVM, brain arteriovenous malformation; CT, computerized tomography; AchA, anterior choroidal artery; PchA, posterior choroidal artery; VA, vertebral artery.

**Figure 5:**
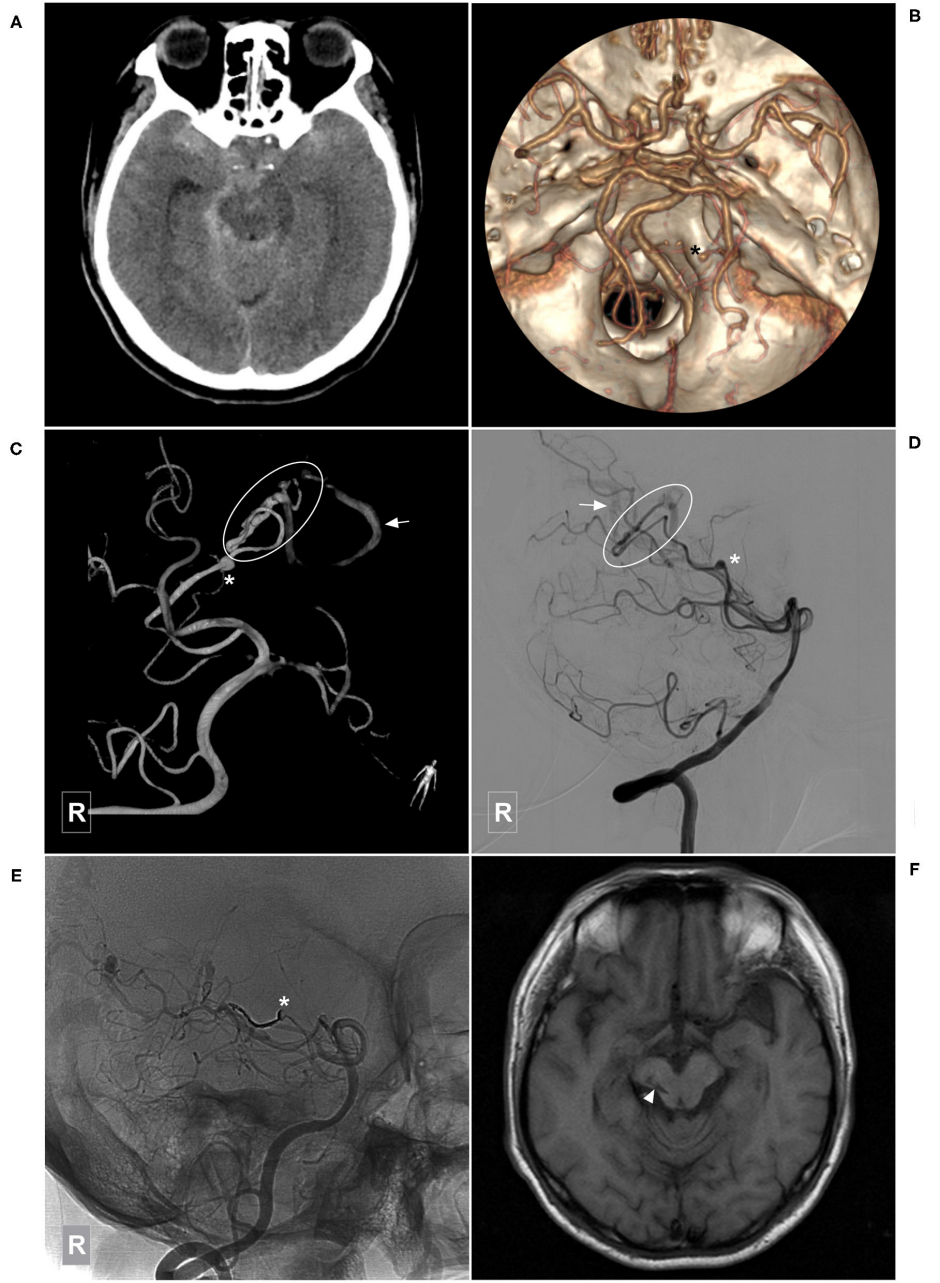
Illustrative case of a Type Ia PCA-BAVM with flow-related aneurysm. **(A)** CT shows diffuse subarachnoid hemorrhage. **(B)** CT angiography shows an aneurysm (asterisk) originated from the right lateral PchA. **(C,D)** Angiogram of the right VA in 3D **(C)** and lateral **(D)** views shows a BAVM (encircled area) supplied by the lateral PchA. Arrow denotes the superficial draining vein and asterisk denotes the aneurysm. **(E)** Unsubtracted angiogram of the right VA shows obliteration of the aneurysm (asterisk) and partial obliteration of the BAVM. **(F)** Magnetic resonance imaging at 3-month follow-up shows an old infarction focus (arrowhead) at the midbrain. BAVM, brain arteriovenous malformation; CT, computerized tomography; PchA, posterior choroidal artery; VA, vertebral artery.

**Figure 6:**
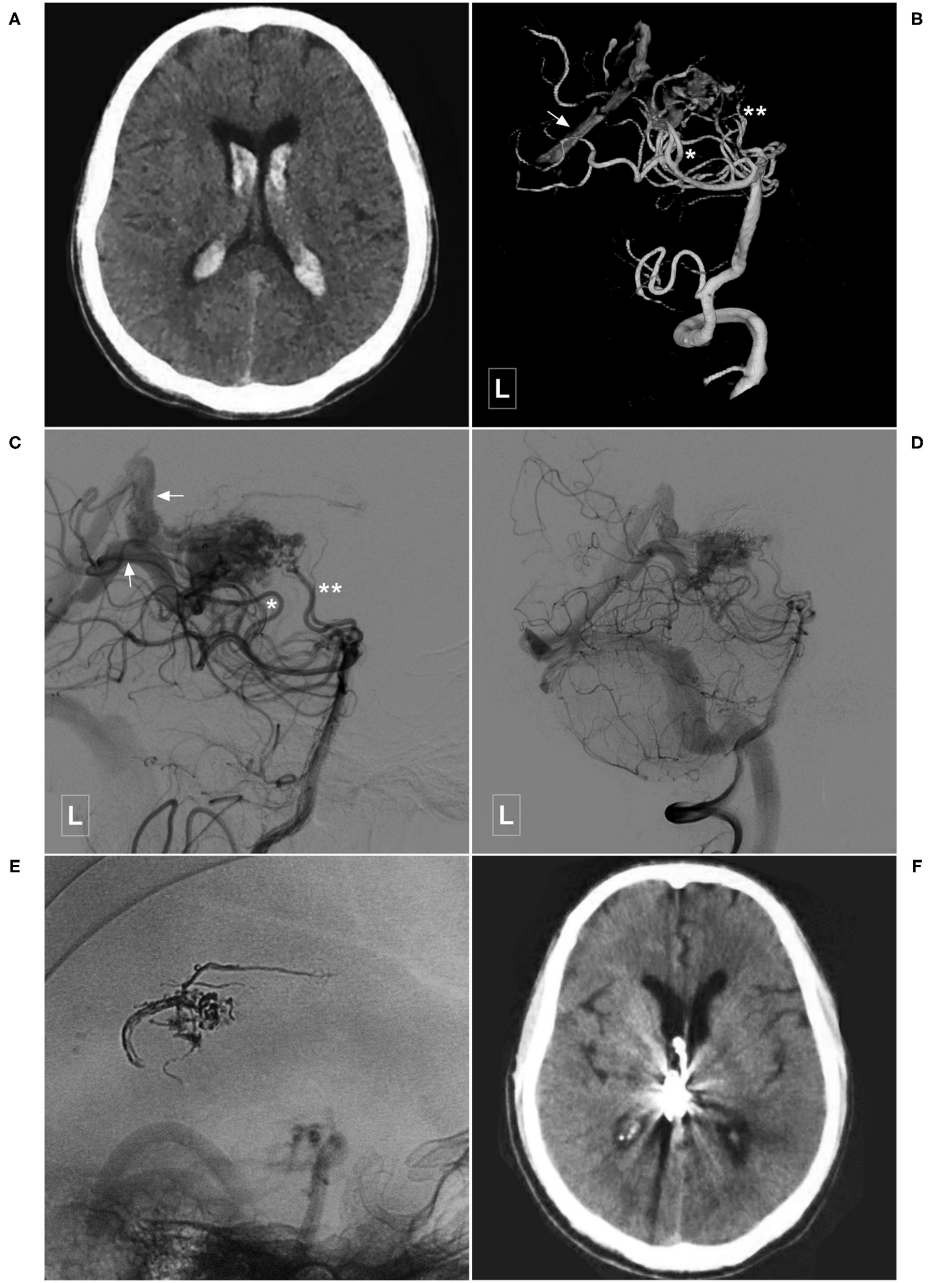
Illustrative case of a Type Ib PCA-BAVM. **(A)** CT shows intraventricular hemorrhage. **(B,C)** Angiogram of the left VA in 3D **(B)** and lateral **(C)** views shows a BAVM supplied by the lateral PchA (single asterisk), and the perforating artery (double asterisks) of the PCA and drained through the deep veins (arrow). **(D,E)** Angiogram of the left VA shows that the BAVM is partially embolized. **(E)** X-ray of the cranium shows the casting Onyx. **(F)**, Follow-up CT shows the intraventricular hemorrhage has resolved. BAVM, brain arteriovenous malformation; CT, computed tomography; PCA, posterior cerebral artery; PchA, posterior choroidal artery; VA, vertebral artery.

**Figure 7:**
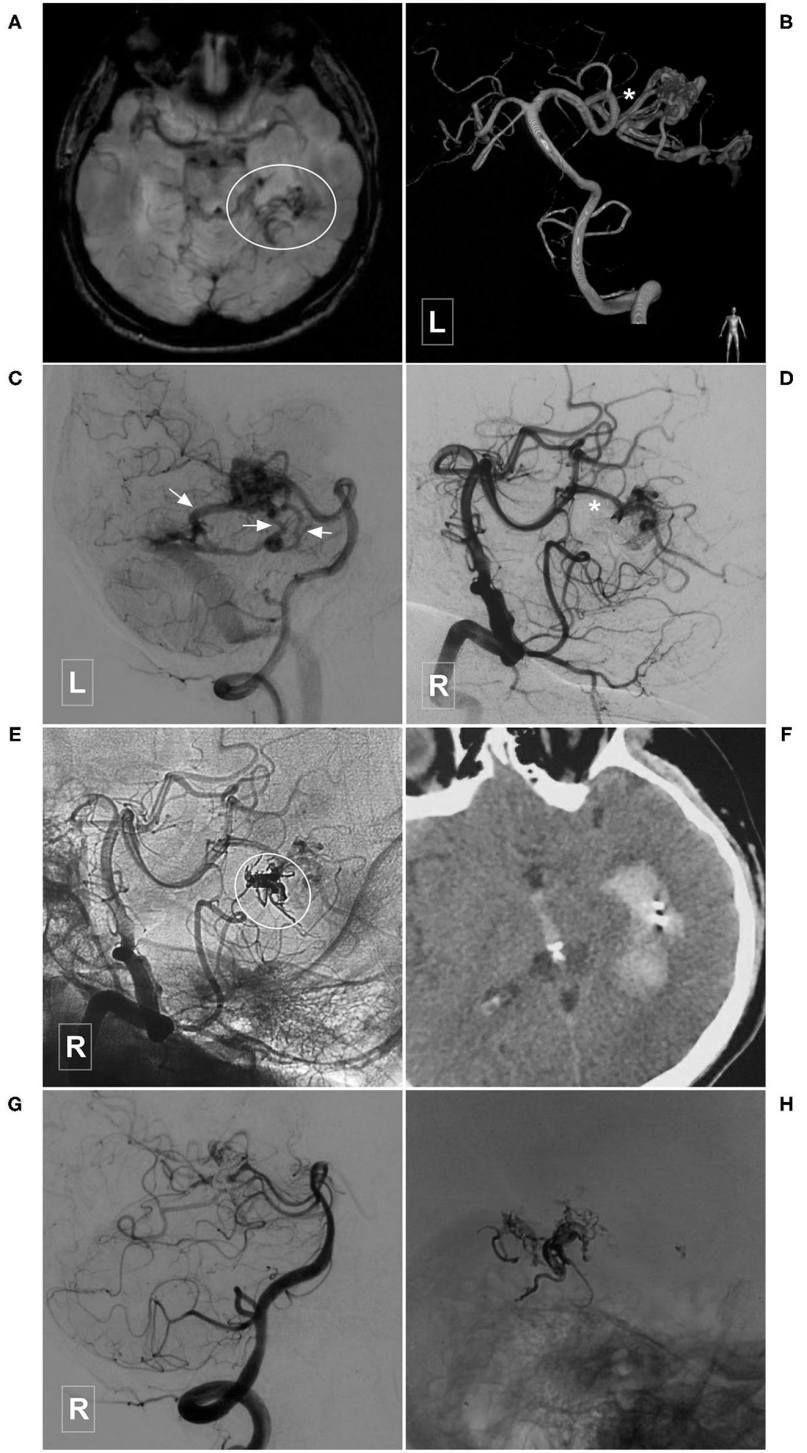
Illustrative case of a Type IIa PCA-BAVM. **(A)**, Magnetic resonance imaging shows flow-voids at the medial temporal lobe (encircled area). **(B)** Angiogram of the left VA in 3D view reveals a BAVM supplied by the temporal branch (asterisk) of the PCA. **(C)** Angiogram of the left VA in lateral view shows that the BAVM drains through multiple superficial veins (arrow). **(D,E)** Subtracted **(D)** and unsubtracted **(E)** angiogram of the right VA shows that the BAVM is partially embolized (encircled area) via the temporal branch (asterisk) of the PCA. **(F)** Computed tomography shows left intraventricular hemorrhage 4 h after the embolization. **(G)**, Follow-up angiogram of the right VA 3 months later shows nearly complete embolization of the BAVM. **(H)**, X-ray of the cranium shows the casting Onyx. BAVM, brain arteriovenous malformation; PCA, posterior cerebral artery; VA, vertebral artery.

**Figure 8:**
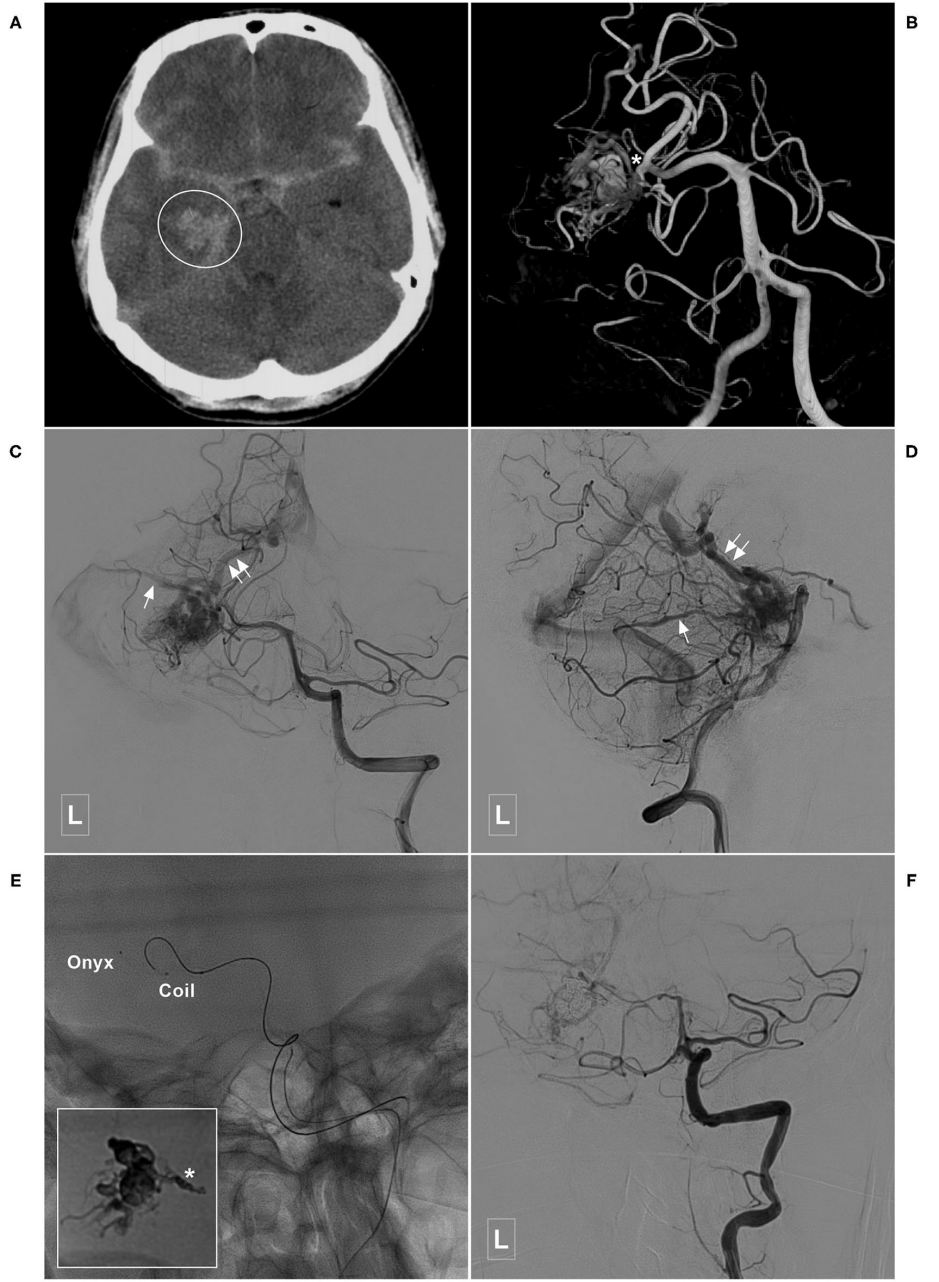
Illustrative case of a Type IIa PCA-BAVM with intranidal aneurysm. **(A)** Computed tomography shows diffuse subarachnoid hemorrhage and a hematoma (encircled area) in the right medial temporal lobe. **(B)** 3D angiogram of the VA shows a BAVM supplied by the temporal branch (asterisk) of the right PCA. An intranidal aneurysm can be seen in the nidus. **(C,D)** Angiogram of the left VA in anteroposterior **(C)** and lateral **(D)** views shows that the BAVM drains both to the superficial (single asterisk) and deep (double asterisks) veins. **(E)** X-ray of the cranium shows the embolization process of the BAVM. One proximal microcatheter is used to release the coils to establish the “pressure cooker” effect. Another distal microcatheter is used to cast the Onyx. Enlarged picture in the square shows the coils (asterisk) and Onyx. **(F)** Angiogram of the left VA shows that the BAVM is completely embolized. BAVM, brain arteriovenous malformation; PCA, posterior cerebral artery; VA, vertebral artery.

**Figure 9:**
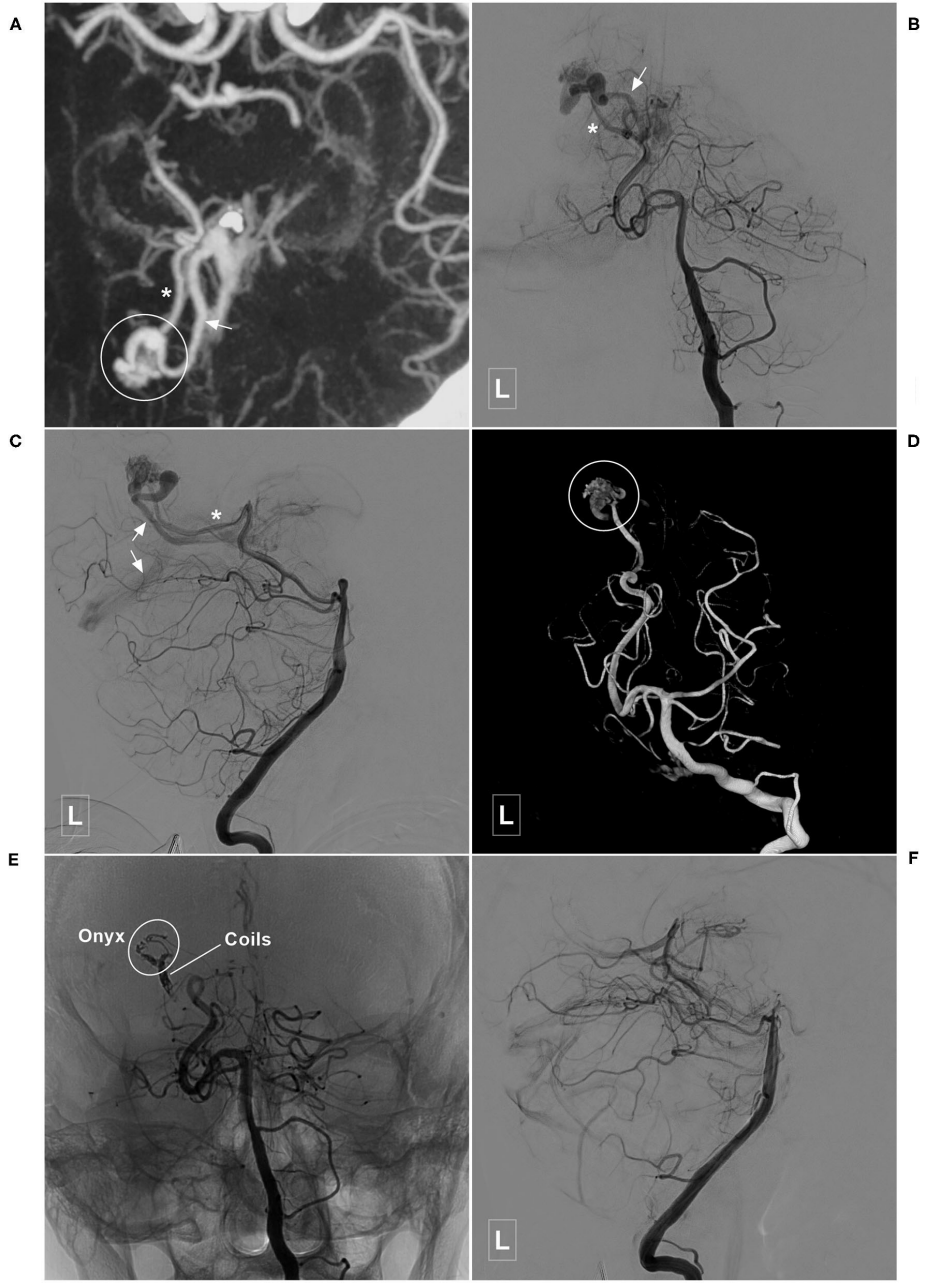
Illustrative case of a Type IIb PCA-BAVM. **(A)** Maximum intensity projection image shows a BAVM (encircled area) supplied by an artery (asterisk) from the occipital branch of the PCA and drained to the deep veins (encircled area). **(B–D)** Angiogram of the left VA in the anteroposterior **(B)**, lateral **(C)**, and 3D **(D)** views shows the feeding artery (asterisk) and deep draining vein (arrow) of the BAVM nidus (encircled area). **(E)** Angiogram of the left VA shows that coils are released proximally to establish a “pressure cooker” effect. Then, the Onyx (encircled area) is Casted. **(F)** Angiogram of the left VA shows that the BAVM is completely embolized. BAVM, brain arteriovenous malformation; PCA, posterior cerebral artery; VA, vertebral artery.

Immediate complete or nearly complete embolization was achieved in 17 (40.5%, 17/42) cases. Partial embolization was achieved in 25 (59.5%, 25/42) cases. Two (4.8%, 2/42) patients experienced intraoperative or postoperative bleeding, all of whom were appropriately managed and experienced satisfactory recovery. Figure 7 illustrates a patient who experienced postoperative bleeding.

The GOS scores at discharge were 3, 4, and 5 in 2 (4.8%, 2/42), 2 (4.8%, 2/42), and 38 (90.4%, 38/42) cases, respectively.

### Follow-Up

Two patients were lost to follow-up. The follow-up period of the remaining 40 patients ranged from 3 to 115 (36.4 ± 23.8) months. Ten patients underwent further radiosurgery or EVT. The GOS scores at the latest follow-up were five and four in 39 patients (97.5%, 39/40) and one patient (2.5%, 1/40), respectively. As many patients refused further angiographic investigation, complete obliteration of the BAVMs was demonstrated in only 17 patients.

### Statistical Analysis

There was no statistical difference between patients in type I and type II groups regarding age, BAVM rupture, SM grade, immediate extent of obliteration, and prognosis (Table 2). Deep venous drainage was more common in patients of the type I group (*P* < 0.001) (Table 3).

**Table 2:** Statistical analysis of the clinical data between type I and Type II PCA-BAVMs.

	**Type I (18 cases)**	**Type II(24 cases)**	***P*-value**
Age	32.3 ± 11.9	30.9 ± 16.7	0.7699
Rupture before admission	15	19	>0.9999
Higher SM grade (≥3)	13	10	0.0653
Complete or nearly complete obliteration	5	12	0.2079
Good prognosis (GOS = 5) at discharge	15	23	0.2972

*GOS, Glasgow Outcome Scale; PCA-BAVM, posterior cerebral artery-brain arteriovenous malformation; SM, Spetzler-Martin*.

**Table 3:** Pattern of venous drainage in type I and type II PCA-BAVMs.

	**Superficial venous drainage alone**	**Concurrent with deep venous drainage**	***P*-value**
I	1 (4.2%)	23 (95.8%)	<0.001
II	12 (66.7%)	6 (33.3%)	

*PCA-BAVM, posterior cerebral artery-brain arteriovenous malformation*.

## Discussion

BAVMs can occur at any part of the brain. A BAVM mainly supplied by the PCA may involve the PchA at the proximal end of the PCA and/or the temporal and occipital branches at the distal end of the PCA. These branches often supply the corpus callosum. The PchA often participates in the choroidal artery system of the posterior ventricle of the brain and can anastomose with the ACA to form a callosal circle (7–10). A case of BAVM mainly supplied by the PchA is illustrated in Figure 4.

Therefore, the blood supply of the PCA-BAVMs is complex. PCA-BAVMs at different locations have different feeding arteries. This study found that a PCA-BAVM supplied by the proximal segment of the PCA was more commonly supplied by the PchA, while a PCA-BAVM supplied by the distal segment of the PCA was mainly supplied by the temporal and occipital branches. Because a BAVM could hijack the feeding artery to the greatest extent, the ends of the ACA can also be involved. As the PCA is close to the deep venous system of Galen, a PCA-BAVM often has deep venous drainage. Among the 42 cases in this study, 29 cases (69%) had deep venous drainage (Table 1).

Surgical resection, EVT, and adjuvant radiotherapy are available treatments for BAVMs. In recent years, EVT has also been used as the main approach for BAVMs, especially for the ruptured ones (11–13). The main aim of EVT is securing the weak structures. A targeted EVT aimed at the ruptured part of the nidus can reduce the risk of early rebleeding (6). As illustrated in Figure 5, embolization of the aneurysm is in priority.

For BAVMs, the SM grading system is most popular for predicting surgical outcome. It categorizes the BAVMs into five grades based on size, existence of deep venous drainage, and eloquence of location (14). In 2010, a supplementary novel Lawton–Young grading system was proposed to predict the treatment outcomes of BAVMs, which is a better predictor of neurological outcome after BAVMs surgery and supplemented the SM system greatly (15).

To our knowledge, no previous study has specifically studied the angioarchitecture of PCA-BAVMs. Therefore, in this study, we classified the PCA-BAVMs into two types according to the blood supply and location. This classification is based on the segmentation of the PCA, which is a reasonable approach. Different types of PCA-BAVMs have different arterial supply, which directly determines the strategy of EVT.

In this study, 42 cases with PCA-BAVMs were analyzed. There were some differences between type I and type II PCA-BAVMs. Type I PCA-BAVMs are mainly supplied by the PchA, the treatment of which was mostly via the PchA. Because the PchA is small, the microcatheter tip can be wedged into a type I PCA-BAVM, with little reflux of liquid embolic material during EVT (Figures 2–4). The type II BAVMs are mainly supplied by the temporal and occipital branches of the PCA. During EVT, the liquid embolic material can easily reflux into the PCA trunk. The “pressure cooker” technique proved to be a useful measure (Figures 8, 9). For cases with additional blood supply from the ACA, further EVT through the ACA can also be tried if EVT through the PCA is difficult or not satisfactory (Figure 6).

EVT is a reasonable option for the PCA-BAVMs. In this study, 97.5% (39/40) of the patients achieved a GOS score of 5 at the last follow-up. Only 2 (4.8%, 2/42) patients experienced intraoperative or postoperative bleeding. Intraoperative bleeding might be caused by the acute increase of intravascular pressure during Onyx casting. In case of intraoperative bleeding, continuous casting of Onyx till obliteration of the bleeding site is the best option. The cause of postoperative bleeding is complex, including early venous occlusion or arterial rupture during microcatheter retrieval. The patient illustrated in Figure 7 was admitted with an unruptured PCA-BAVM. The intraoperative process was unremarkable. The draining veins were patent after embolization. However, the patient experienced sudden onset of headache 4 h after EVT. Head CT revealed IVH. We speculated that the postoperative bleeding might be caused by increased pressure in the vessels near the embolized nidus due to blood flow redistribution.

In addition, we also compared the angiographic and clinical characteristics between the patients with type I and type II PCA-BAVMs. No statistical significance was noted between the two groups in age, rupture before admission, SM grade, extent of obliteration, and prognosis. Type I PCA-BAVMs were prone to have deep venous drainage (Tables 2, 3). Type Ia PCA-BAVMs are the easiest type to treat due to the single blood supply. However, it becomes more difficult to treat for type Ib PCA-BAVMs due to the multiple sources of feeding arteries. Type II PCA-BAVMs are supplied by the distal branches of the PCA and EVT is relatively easy. However, care should be taken during EVT to prevent the liquid embolic material reflux into the proximal PCA trunk. Therefore, our classification of PCA-BAVMs based on the feeding arteries can effectively guide the EVT.

## Limitations

This is a retrospective study with limited sample size, and the conclusion in this study should be cautiously interpreted. The rate of angiographic follow-up in this study is low, which makes it difficult to evaluate the long-term efficacy of EVT. As EVT was given priority for the management of BAVMs in our center, comparison with other treatments could not be performed in this study.

## Data Availability Statement

The raw data supporting the conclusions of this article will be made available by the authors, without undue reservation.

## Ethics Statement

The studies involving human participants were reviewed and approved by the ethics committee of The First Hospital of Jilin University. Written informed consent for participation was not required for this study in accordance with the national legislation and the institutional requirements. Written informed consent was obtained from the individual(s) for the publication of any potentially identifiable images or data included in this article.

## Author Contributions

JY: contributed to the conception and design of the manuscript and critically revised the manuscript. KH and CL: wrote the manuscript. CL and HS: collected the medical records of the patients. All authors approved the final version of this manuscript.

## Conflict of Interest

The authors declare that the research was conducted in the absence of any commercial or financial relationships that could be construed as a potential conflict of interest.

## References

[1] Lawton MT Rutledge WC Kim H Stapf C Whitehead KJ Li DY. Brain arteriovenous malformations. Nat Rev Dis Primers. (2015) 1:15008. 10.1038/nrdp.2015.8 27188382

[2] Solomon RA Connolly ES Jr. Arteriovenous malformations of the brain. N Engl J Med. (2017) 376:1859–66. 10.1056/NEJMra1607407 28489992

[3] Piao J Ji T Guo Y Xu K Yu J. Brain arteriovenous malformation with transdural blood supply: current status. Exp Ther Med. (2019) 18:2363–8. 10.3892/etm.2019.7731 31555346 PMC6755268

[4] Ecker RD. Epistemology of brain arteriovenous malformations. World Neurosurg. (2016) 89:697–8. 10.1016/j.wneu.2015.11.035 26679261

[5] Batista LL Azevedo HC. Anterior cerebral artery. J Neurosurg. (2004) 101:717. 10.1016/B0-44-306600-0/50010-9 15481734

[6] Hou K Xu K Chen X Ji T Guo Y Yu J. Targeted endovascular treatment for ruptured brain arteriovenous malformations. Neurosurg Rev. (2020) 43:1509–18. 10.1007/s10143-019-01205-1 31720915

[7] Hou K Li G Luan T Xu K Yu J. The prospects and pitfalls in the endovascular treatment of moyamoya disease-associated intracranial aneurysms. Neurosurg Rev. (2020). 10.1007/s10143-020-01261-y. [Epub ahead of print]. 32052219

[8] Yu J Xu N Zhao Y Yu J. Clinical importance of the anterior choroidal artery: a review of the literature. Int J Med Sci. (2018) 15:368–75. 10.7150/ijms.22631 29511372 PMC5835707

[9] Luo Q Wang H Xu K Yu J. Endovascular treatments for distal posterior cerebral artery aneurysms. Turk Neurosurg. (2012) 22:141–7. 10.5137/1019-5149.JTN.4079-11.0 22437286

[10] Robert T Ciccio G Sylvestre P Chiappini A Weil AG Smajda S. Anatomic and angiographic analyses of ophthalmic artery collaterals in moyamoya disease. AJNR Am J Neuroradiol. (2018) 39:1121–6. 10.3174/ajnr.A5622 29650781 PMC7410630

[11] Baharvahdat H Blanc R Fahed R Pooyan A Mowla A Escalard S. Endovascular treatment as the main approach for Spetzler-Martin grade III brain arteriovenous malformations. J Neurointerv Surg. (2020). 10.1136/neurintsurg-2020-016450. [Epub ahead of print]. 32989031

[12] Mosimann PJ Chapot R. Contemporary endovascular techniques for the curative treatment of cerebral arteriovenous malformations and review of neurointerventional outcomes. J Neurosurg Sci. (2018) 62:505–13. 10.23736/S0390-5616.18.04421-1 29582979

[13] Iosif C Mendes GA Saleme S Ponomarjova S Silveira EP Caire F. Endovascular transvenous cure for ruptured brain arteriovenous malformations in complex cases with high Spetzler-Martin grades. J Neurosurg. (2015) 122:1229–38. 10.3171/2014.9.JNS141714 25794338

[14] Spetzler RF Martin NA. A proposed grading system for arteriovenous malformations. J Neurosurg. (1986) 65:476–83. 10.3171/jns.1986.65.4.0476 3760956

[15] Lawton MT Kim H McCulloch CE Mikhak B Young WL. A supplementary grading scale for selecting patients with brain arteriovenous malformations for surgery. Neurosurgery. (2010) 66:702–13. 10.1227/01.NEU.0000367555.16733.E1 20190666 PMC2847513

